# Supplementary material for: The effect of drop-in centers on access to HIV testing, case finding, and condom use among female sex workers in Addis Ababa, Ethiopia
Source: PeerJ. 2023 Oct 17;11:e16144. doi: 10.7717/peerj.16144 (PMC10588723; doi:10.7717/peerj.16144)

Table 1: Mantel-Haenszel bounds result for lifetime test to know HIV status


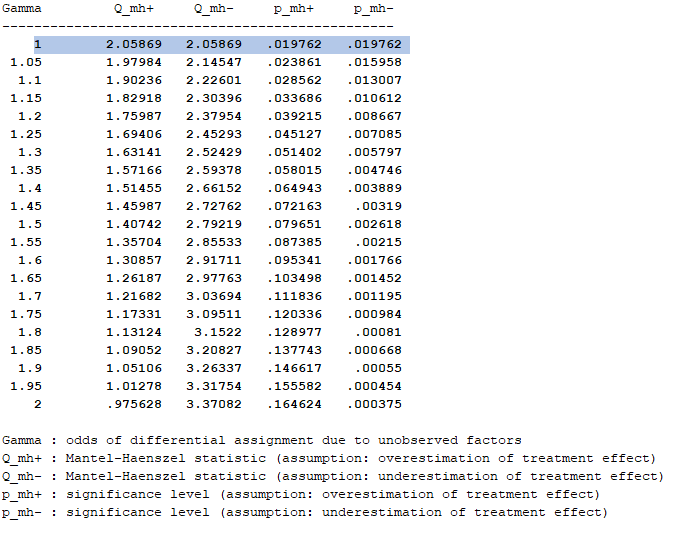


Table 2: Mantel-Haenszel bounds result for the outcome finding HIV positives


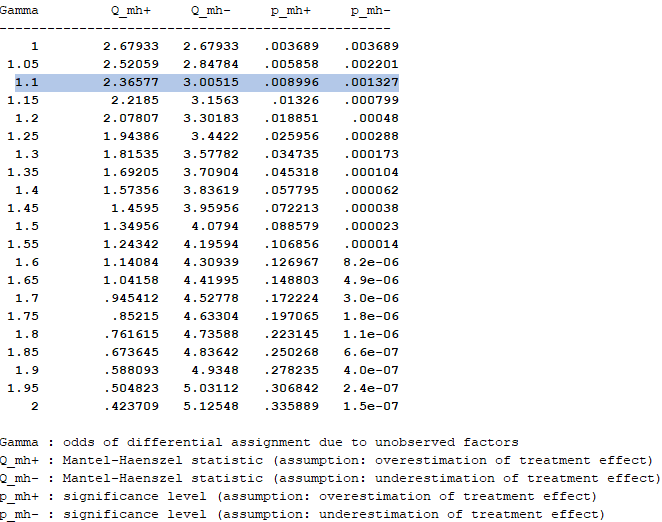


Table 3: Mantel-Haenszel bounds result for aware of their HIV positive status


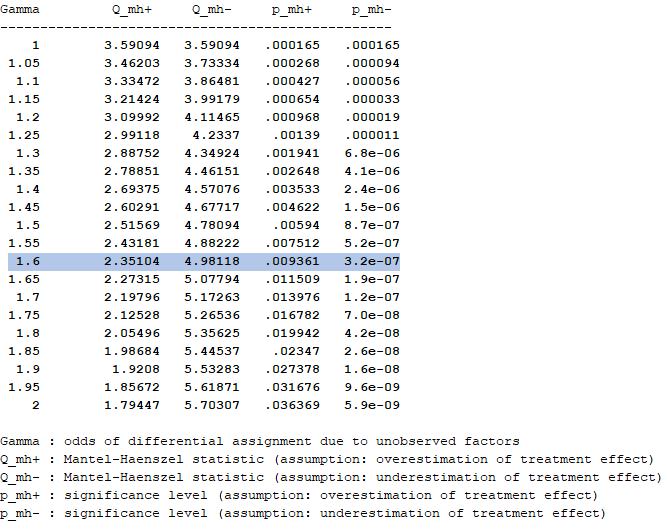


Table 4: Mantel-Haenszel bounds result for consistent condom use


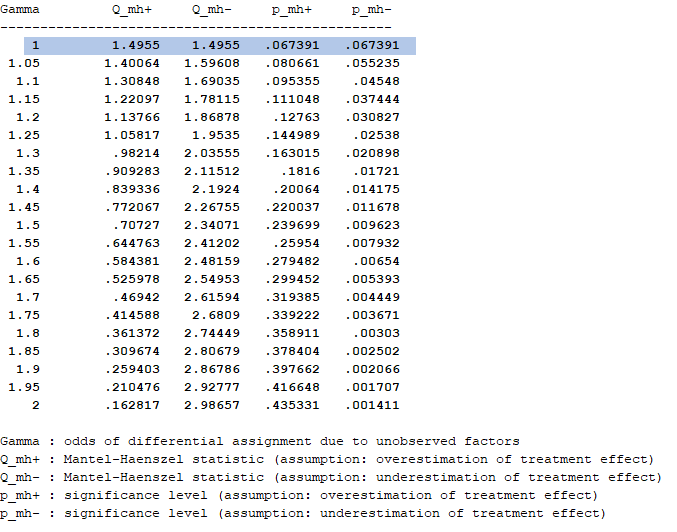

Supplement: Supplemental Information 1 — Mantel-Haenszel bounds result for lifetime test to know HIV status, finding HIV positives, aware of their HIV positive status, and consistent condom use. [file peerj-11-16144-s001.docx]
