# Supplementary figures and images for: The effect of drop-in centers on access to HIV testing, case finding, and condom use among female sex workers in Addis Ababa, Ethiopia
Source: PeerJ. 2023 Oct 17;11:e16144. doi: 10.7717/peerj.16144 (PMC10588723; doi:10.7717/peerj.16144)

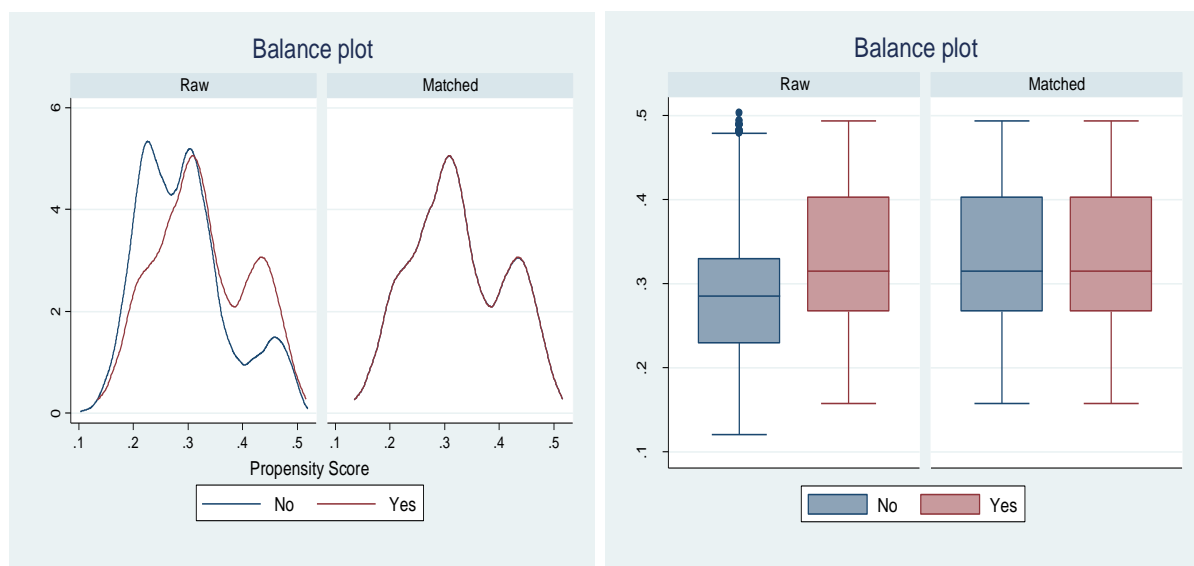

Fig 1: Propensity score density and box balance plot of FSWs in Addis Ababa, 2020

Supplement: Supplemental Information 2 — All the covariates were balanced after propensity score matching was applied. [file peerj-11-16144-s002.pdf]
